# Procedure-based severity index for inpatients: development and validation using administrative database

**DOI:** 10.1186/s12913-015-0889-x

**Published:** 2015-07-08

**Authors:** Hayato Yamana, Hiroki Matsui, Kiyohide Fushimi, Hideo Yasunaga

**Affiliations:** Department of Clinical Epidemiology and Health Economics, School of Public Health, The University of Tokyo, 7-3-1 Hongo, Bunkyo-ku, Tokyo, 113-0033 Japan; Bunkyo City Public Health Center, 1-16-21 Kasuga, Bunkyo-ku, Tokyo, 112-8555 Japan; Department of Health Policy and Informatics, Tokyo Medical and Dental University Graduate School of Medicine, 1-5-45 Yushima, Bunkyo-ku, Tokyo, 113-8510 Japan

**Keywords:** Administrative data, Mortality, Risk adjustment, Severity

## Abstract

**Background:**

Risk adjustment is important in studies using administrative databases. Although utilization of diagnostic and therapeutic procedures can represent patient severity, the usability of procedure records in risk adjustment is not well-documented. Therefore, we aimed to develop and validate a severity index calculable from procedure records.

**Methods:**

Using the Japanese nationwide Diagnosis Procedure Combination database of acute-care hospitals, we identified patients discharged between 1 April 2012 and 31 March 2013 with an admission-precipitating diagnosis of acute myocardial infarction, congestive heart failure, acute cerebrovascular disease, gastrointestinal hemorrhage, pneumonia, or septicemia. Subjects were randomly assigned to the derivation cohort or the validation cohort. In the derivation cohort, we used multivariable logistic regression analysis to identify procedures performed on admission day which were significantly associated with in-hospital death, and a point corresponding to regression coefficient was assigned to each procedure. An index was then calculated in the validation cohort as sum of points for performed procedures, and performance of mortality-predicting model using the index and other patient characteristics was evaluated.

**Results:**

Of the 539 385 hospitalizations included, 270 054 and 269 331 were assigned to the derivation and validation cohorts, respectively. Nineteen significant procedures were identified from the derivation cohort with points ranging from −3 to 23, producing a severity index with possible range of −13 to 69. In the validation cohort, c-statistic of mortality-predicting model was 0.767 (95 % confidence interval: 0.764–0.770). The ω-statistic representing contribution of the index relative to other variables was 1.09 (95 % confidence interval: 1.03–1.17).

**Conclusions:**

Procedure-based severity index predicted mortality well, suggesting that procedure records in administrative database are useful for risk adjustment.

**Electronic supplementary material:**

The online version of this article (doi:10.1186/s12913-015-0889-x) contains supplementary material, which is available to authorized users.

## Background

Risk adjustment is an important component in clinical epidemiology and health services research using administrative databases, but its methods remain controversial. Administrative databases are widely used in studies because of their availability and large sample sizes, and risk-adjusted mortality is employed as one of the outcome measures. However, the validity of risk-adjustment models for administrative data has been questioned repeatedly [1–4]. It has been argued that administrative data lack important clinical information [5–8] and often do not make distinctions between conditions present on admission and complications occurring during hospitalization [6–10]. Inadequate risk adjustment can lead to misleading consequences such as confounding by indications and low rating of facilities that care for sicker patients. Thus, appropriate risk-adjustment models are desired.

Previous studies have shown that the performance of risk-adjustment models using administrative databases improves when detailed clinical information is added. In addition to patients’ demographic characteristics, comorbid illnesses recorded in administrative data enabled risk adjustment using measures such as the Charlson comorbidity index (CCI) [11]. Furthermore, models using laboratory data, vital signs, and other clinical findings provided better predictions of mortality [12–15], and models using disease-specific diagnostic tests and treatments have been introduced for some diseases [16–19]. Meanwhile, precise laboratory and clinical data are not available in most administrative databases. Therefore, an alternative method has been reported, in which surgeries and major therapeutic procedures are associated with in-hospital death [20].

In addition to major therapeutic procedures, commonly performed procedures, diagnostic or therapeutic, can reflect the severity of patients on admission. For example, patients who receive oxygen therapy are expected to be in a severe condition compared with those who do not. However, there have been no evaluations of risk-adjustment models that use commonly performed procedures. In addition, previous models using laboratory and clinical data were developed and validated in limited regions.

The aims of the present study were to develop an index of severity using procedure records in a nationwide database, and to examine the ability of this index to predict in-hospital death.

## Methods

### Data source

The Diagnosis Procedure Combination database is a national administrative database of acute-care inpatients in Japan that is linked with a payment system. The mandatory-participating academic hospitals (all 82 hospitals) and voluntary-participating community hospitals provide claims data of all of their acute-care inpatients. In 2012, there were approximately 1,000 participating hospitals with 7 million admissions recorded annually, representing 50 % of all acute-care hospitalizations in Japan.

The database includes the following data: hospital identification code; patient demographics; diagnoses; admission and discharge status; surgeries and procedures performed; drugs used; and special reimbursements for specific conditions. Up to 12 diagnoses for each admission are recorded, and coded using the *International Classification of Diseases,* Tenth Revision (ICD-10). One diagnosis each is recorded for “main diagnosis,” “admission-precipitating diagnosis,” “most resource-consuming diagnosis,” and “second most resource-consuming diagnosis.” A maximum of four diagnoses each are recorded for “comorbidities present on admission” and “conditions arising after admission.” Suspected diagnoses are allowed to be recorded, in which case they are designated as such. Surgeries, drugs, procedures, and special reimbursements are coded according to the Japanese fee schedule for reimbursement [21], and their dates of use or application are recorded. The daily quantities of each drug administered are also recorded.

### Study cohort

We included all adult patients (≥18 years) discharged between 1 April 2012 and 31 March 2013 with a confirmed admission-precipitating diagnosis of acute myocardial infarction, congestive heart failure, acute cerebrovascular disease, gastrointestinal hemorrhage, pneumonia, or septicemia. The identification of these six diseases was based on the Classifications Software for Mortality Reporting developed by the Healthcare Cost and Utilization Project [22], and the following Classifications Software categories were used for the six diseases, respectively: 100, 108, 109, 153, 122, and 2. For congestive heart failure, we also included hypertensive heart disease with heart failure (ICD-10 code: I11.0, I13.0, or I13.2). We excluded the following patients based on their information on the day of admission: those who were admitted to intensive care unit (including coronary care unit); and those who received cardiopulmonary life support (cardiopulmonary resuscitation, electrical cardioversion, cardiopulmonary bypass, extracorporeal membrane oxygenation, or ventricular assist device). We identified the former using reimbursement information, and the latter using procedure information.

The data for diagnostic and therapeutic procedures performed on the day of admission, use of catecholamines (epinephrine, norepinephrine, dopamine, and dobutamine) and vasopressin on the day of admission, and use of blood transfusions (red blood cells, platelets, fresh frozen plasma, and albumin) on the day of admission were extracted. A list of the procedures and codes examined in this study is shown in the Additional file 1. For the examinations, examples of the tested items are also listed. Patients who underwent at least one procedure categorized under a given code were assigned that specific code. For example, “D007, blood chemistry tests” would be coded for patients who underwent creatinine testing, as well as for patients who underwent sodium, potassium, and chloride testing. Comorbidities were examined using the diagnoses recorded as comorbidities present on admission, and CCI values were calculated using the coding algorithm [23] and weight assignment [24] reported by Quan et al.

We randomly assigned the eligible patients to the derivation cohort or validation cohort. We developed the severity index for inpatients using the derivation cohort, and tested its performance in the validation cohort.

### Index development

In the derivation cohort, we first examined the proportion of patients who underwent each procedure (including use of catecholamines and vasopressin) on the day of admission. For each procedure with ≥1 % prevalence, the chi-square test was used to evaluate the association with in-hospital death. The procedures positively associated with in-hospital death (*P* < 0.1) were retained for further analysis. Procedures with a correlation (phi coefficient >0.6) were managed in the following manner: (i) a group of procedures usually performed simultaneously were combined into a single variable as at least one procedure; and (ii) for a group of procedures performed consecutively, only the procedure usually performed first was retained. Subsequently, a logistic regression model was developed with in-hospital death as the outcome variable. In the model, the admission-precipitating diagnosis, age, sex, and CCI were included as categorical covariates (age categories: <60, 60–69, 70–79, 80–89, ≥90; CCI categories: 0, 1, 2, ≥3) in addition to the procedures.

Using the statistically significant (*P* < 0.05) regression coefficients obtained with the model, we derived an index-calculating formula by the method of Sullivan et al. [25], using CCI = 1 as a reference. Specifically, a point was assigned to each procedure so that it equaled the integer nearest to the quotient of the regression coefficient for the procedure divided by the regression coefficient for CCI = 1. Thus, the points for each procedure were derived to represent the effect on death relative to the CCI. The severity index for each patient could then be calculated as the sum of the points assigned to the procedures performed on the patient.

### Index validation

The severity index was calculated for patients in the validation cohort. We examined the distribution of its values, and used a logistic regression model with the index as a continuous variable (model 1) to examine its association with in-hospital death. For every value, the expected death rate among patients with the value was compared with the observed death rate.

We then constructed multiple logistic regression models with different independent variables: severity index, diagnosis, age, and sex (model 2); diagnosis, age, sex, and CCI (model 3); severity index, diagnosis, age, sex, and CCI (model 4). The discriminatory abilities of the different models were assessed using the *c*-statistics. We used the integrated discrimination improvement (IDI) [26] to evaluate the improvement of model discrimination by adding the severity index. The IDI is a difference in the discrimination slope (difference between the mean predicted probability of an event for those with events and the corresponding mean predicted probability of an event for those without events) between two models and is a measure of the improvement in model performance. In this study, the IDI was calculated for a comparison of model 4 with model 3.

We evaluated the relative contribution of the severity index to the prediction of death using the *ω*-statistic [27]. The *ω*-statistic is the ratio of the variances of the contributions of two groups of variables to the log-odds of the outcome in a logistic regression model. In this study, we used model 4, and compared the relative contribution of the severity index with that of four other variables. In addition, the calibration of model 4 was evaluated using the Hosmer–Lemeshow decile partition.

We conducted further analyses to test the performance of the severity index across various subgroups of patients. Using the severity index derived from all patients in the derivation cohort, model 4 was constructed for the following subgroups of the validation cohort: those who arrived in an ambulance and those who did not; those who were referred by another institution and those with no referral. We also built models for each admission-precipitating diagnosis, with severity index, age, sex, and CCI as independent variables. The model discrimination and calibration were evaluated for each subgroup.

The *P* values were 2 sided. Statistical analyses were performed using IBM SPSS for Windows, version 22.0 (IBM Corp., Armonk, NY, USA). Because of the anonymous nature of the data, the need for informed consent was waived. Study approval was obtained from the Institutional Review Board of The University of Tokyo.

## Results

### Patient characteristics

We identified 604,579 adult patients with one of the six diseases as the admission-precipitating diagnosis during the study period. Of these patients, 65,194 were excluded because of intensive care unit admission (*n* = 59,995) or cardiopulmonary life support (*n* = 7,019) on the day of admission, leaving 539,385 patients for analysis. For these patients, the mean age was 74.1 years, 57.5 % were male, and the in-hospital mortality rate was 9.5 %. The background characteristics of all patients, patients assigned to the derivation cohort (*n* = 270,054), and patients assigned to the validation cohort (*n* = 269,331) are presented in Table 1. The characteristics were similar between the two cohorts.

**Table 1:** Characteristics of patients included in the study

	Derivation cohort		Validation cohort		All patients	
Total number	270 054		269 331		539 385	
Admission-precipitating diagnosis, n (%)						
Acute myocardial infarction	10 421 (3.9)		10 176 (3.8)		20 597 (3.8)	
Congestive heart failure	56 970 (21.1)		56 927 (21.1)		113 897 (21.1)	
Acute cerebrovascular disease	89 983 (33.3)		90 071 (33.4)		180 054 (33.4)	
Gastrointestinal hemorrhage	35 073 (13.0)		34 429 (12.8)		69 502 (12.9)	
Pneumonia	68 387 (25.3)		68 386 (25.4)		136 773 (25.4)	
Septicemia	9220 (3.4)		9342 (3.5)		18 562 (3.4)	
Male, n (%)	155 273 (57.5)		154 857 (57.5)		310 130 (57.5)	
Age, mean (SD)	74.1 (14.2)		74.1 (14.2)		74.1 (14.2)	
Age by category, n (%)						
<60	37 363 (13.8)		37 425 (13.9)		74 788 (13.9)	
60–69	47 235 (17.5)		46 833 (17.4)		94 068 (17.4)	
70–79	73 468 (27.2)		73 509 (27.3)		146 977 (27.2)	
80–89	84 965 (31.5)		84 964 (31.5)		169 929 (31.5)	
≥90	27 023 (10.0)		26 600 (9.9)		53 623 (9.9)	
Charlson comorbidity index, n (%)						
0	162 038 (60.0)		161 597 (60.0)		323 635 (60.0)	
1	34 307 (12.7)		34 015 (12.6)		68 322 (12.7)	
2	52 841 (19.6)		52 688 (19.6)		105 529 (19.6)	
≥3	20 868 (7.7)		21 031 (7.8)		41 899 (7.8)	
In-hospital death, n (%)	25 638 (9.5)		25 449 (9.4)		51 087 (9.5)	
Length of stay in days, median (IQR)	15 (9–29)		15 (9–29)		15 (9–29)	

IQR – interquartile range

SD – standard deviation

### Index development

There were 38 procedures with ≥1 % prevalence in the derivation cohort. Their rate of use in the surviving and deceased patients, and the results of chi-square tests are presented in Table 2. Of the 28 procedures significantly associated with in-hospital death, 11 were correlated with each other in five groups. The three blood examinations (blood chemistry, hematology, plasma protein immunology), two urine examinations (general urine test, urine microscopy), and two microbiological examinations (bacterial microscopy, bacterial culture) were combined as blood tests (excluding coagulation), urinalyses (excluding chemistry), and bacterial microscopy or culture, respectively. Thus, for example, “blood tests (excluding coagulation)” would be counted for patients who underwent sodium, potassium, and hemoglobin testing, as well as for patients who underwent C-reactive protein testing. Central venous infusion and oxygen administration were respectively correlated with central venous catheter insertion and pulse oximetry, which logically precede each procedure. Therefore, central venous catheter insertion and pulse oximetry were retained, while central venous infusion and oxygen administration were excluded from the analysis. The 22 candidate variables were then entered into a logistic regression model, the results of which are presented in Table 3. There were seven procedure variables significantly associated with decreased risk of death, and twelve procedure variables significantly associated with increased risk of death. Because CCI = 1 was not significantly associated with increased odds of death, we divided the regression coefficient for CCI = 2 (0.182) by two to obtain an estimate for the coefficient for CCI = 1 and used it as a reference. The points assigned to each procedure are also presented in Table 3.

**Table 2:** Procedures with ≥1 % prevalence in the derivation cohort

	Surviving patients (n = 244 416)		Deceased patients (n = 25 638)		*P* value^a^	
	%	(n)	%	(n)		
Blood chemistry tests	87.2	(213 046)	89.5	(22 949)	<0.001	
Hematology tests	86.1	(210 402)	88.6	(22 704)	<0.001	
Plasma protein immunology tests^b^	81.0	(198 064)	85.4	(21 896)	<0.001	
Radiography	78.1	(190 981)	81.6	(20 922)	<0.001	
Electrocardiogram	69.3	(169 490)	69.7	(17 869)	0.244	#
Peripheral intravenous infusion	65.1	(159 043)	72.5	(18 581)	<0.001	
Coagulation tests	59.5	(145 418)	62.2	(15 937)	<0.001	
Computed tomography scan	50.6	(123 559)	62.2	(15 938)	<0.001	
Infectious disease immunology tests^c^	50.5	(123 481)	49.2	(12 622)	<0.001	†
Hepatitis virus tests	39.5	(96 552)	37.8	(9681)	<0.001	†
Heart rate/respiration monitoring	37.4	(91 457)	49.8	(12 770)	<0.001	
Pulse oximetry	34.9	(85 254)	51.2	(13 128)	<0.001	
Oxygen administration	30.5	(74 526)	54.5	(13 961)	<0.001	
Urine tests (general)	32.0	(78 225)	35.3	(9046)	<0.001	
Endocrinology tests	28.3	(69 282)	32.1	(8222)	<0.001	
Bacterial culture	25.0	(61 069)	34.5	(8852)	<0.001	
Urine microscopy	21.6	(52 891)	24.2	(6211)	<0.001	
Immunohematology tests^d^	21.8	(53 197)	21.4	(5486)	0.175	#
Bacterial microscopy	20.1	(49 180)	27.8	(7127)	<0.001	
Urinary catheter insertion	18.0	(43 933)	37.2	(9534)	<0.001	
Ultrasound imaging	18.9	(46 118)	18.8	(4830)	0.909	#
Magnetic resonance imaging	18.6	(45 489)	10.2	(2615)	<0.001	†
Acid-fast bacilli culture	8.5	(20 879)	10.6	(2727)	<0.001	
Bacterial drug susceptibility tests	7.2	(17 667)	12.7	(3267)	<0.001	
Sputum suction	5.3	(13 061)	22.2	(5680)	<0.001	
Use of catecholamines or vasopressin	5.1	(12 371)	12.3	(3143)	<0.001	
Red blood cell transfusion	5.0	(12 268)	5.2	(1324)	0.313	#
Nucleic acid amplification tests	3.7	(9102)	4.6	(1178)	<0.001	
Tumor markers	3.3	(8018)	2.6	(667)	<0.001	†
Invasive arterial pressure measurement	2.3	(5649)	4.4	(1130)	<0.001	
Autoantibody tests	2.3	(5740)	1.9	(488)	<0.001	†
Urine chemistry tests	2.1	(5130)	2.6	(654)	<0.001	
Gastric drainage tube insertion	1.6	(3993)	6.1	(1559)	<0.001	
Central venous catheter insertion	1.2	(2997)	5.3	(1346)	<0.001	
Intratracheal intubation	0.9	(2252)	7.7	(1963)	<0.001	
Stool tests	1.4	(3377)	1.2	(311)	0.027	†
Central venous infusion	1.0	(2467)	4.5	(1163)	<0.001	
Temporary urinary catheterization	1.0	(2465)	1.4	(352)	<0.001	

^a^The *P* values were obtained by the chi-square test for in-hospital death

^b^For example, C-reactive protein, complement activity

^c^For example, viral antibody tests, β-D-glucan assay

^d^For example, blood type test, Coombs test

# Not significant

† Significantly associated with decreased risk of death

**Table 3:** Result of logistic regression analysis in the derivation cohort and points assigned to each procedure

Variable	β	exp(β)	95 % CI	*P* value	Points for procedure
Admission-precipitating diagnosis					
Acute myocardial infarction	Ref				
Congestive heart failure	−0.10	0.90	0.83 – 0.98	0.012	
Acute cerebrovascular disease	−0.11	0.90	0.82 – 0.97	0.010	
Gastrointestinal hemorrhage	−0.51	0.60	0.55 – 0.66	<0.001	
Pneumonia	0.04	1.04	0.95 – 1.13	0.405	
Septicemia	0.73	2.07	1.87 – 2.28	<0.001	
Sex (female)	−0.12	0.89	0.86 – 0.91	<0.001	
Age					
<60	Ref				
60–69	0.35	1.43	1.33 – 1.53	<0.001	
70–79	0.68	1.98	1.86 – 2.11	<0.001	
80–89	1.14	3.12	2.94 – 3.31	<0.001	
≥90	1.60	4.96	4.65 – 5.30	<0.001	
Charlson comorbidity index					
0	Ref				
1	0.03	1.03	0.98 – 1.07	0.233	
2	0.18	1.20	1.16 – 1.24	<0.001	
≥3	0.72	2.05	1.96 – 2.14	<0.001	
Procedure					
Invasive arterial pressure measurement	−0.30	0.74	0.68 – 0.81	<0.001	−3
Blood tests (excluding coagulation)	−0.26	0.77	0.73 – 0.81	<0.001	−3
Radiography	−0.20	0.82	0.79 – 0.86	<0.001	−2
Urinalyses (excluding chemistry)	−0.18	0.84	0.81 – 0.87	<0.001	−2
Temporary urinary catheterization	−0.18	0.84	0.74 – 0.94	0.004	−1
Endocrinology tests	−0.06	0.94	0.91 – 0.97	<0.001	−1
Bacterial microscopy or culture	−0.05	0.95	0.91 – 0.99	0.019	−1
Acid-fast bacilli culture	−0.04	0.96	0.91 – 1.02	0.206	#
Coagulation tests	−0.03	0.97	0.94 – 1.01	0.117	#
Nucleic acid amplification tests	0.01	1.01	0.93 – 1.09	0.842	#
Bacterial drug susceptibility tests	0.06	1.06	1.01 – 1.12	0.022	1
Urine chemistry tests	0.10	1.10	1.00 – 1.21	0.041	1
Heart rate/respiration monitoring	0.18	1.20	1.16 – 1.24	<0.001	2
Peripheral intravenous infusion	0.18	1.20	1.16 – 1.24	<0.001	2
Computed tomography scan	0.25	1.28	1.24 – 1.32	<0.001	3
Pulse oximetry	0.29	1.33	1.29 – 1.37	<0.001	3
Gastric drainage tube insertion	0.32	1.38	1.27 – 1.50	<0.001	4
Urinary catheter insertion	0.47	1.60	1.55 – 1.66	<0.001	5
Central venous catheter insertion	0.49	1.63	1.49 – 1.77	<0.001	5
Use of catecholamines or vasopressin	0.70	2.02	1.92 – 2.13	<0.001	8
Sputum suction	1.11	3.04	2.92 – 3.16	<0.001	12
Intratracheal intubation	2.05	7.77	7.14 – 8.45	<0.001	23

# No points assigned because of non-significance of the coefficient

CI – confidence interval

### Index performance

**Fig. 1:**
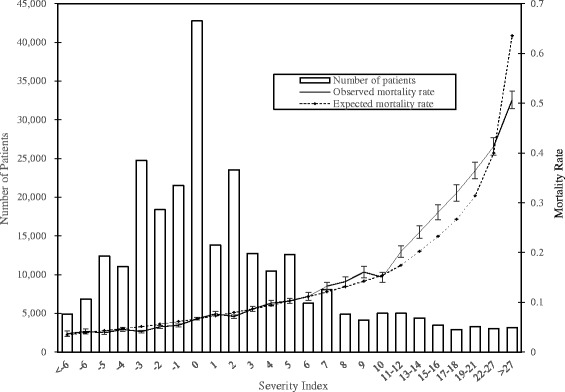
Distribution of the severity index and observed and expected death rates in the validation cohort. The box plots indicate the observed mortality rates and their 95 % confidence intervals

The severity index calculated for patients in the validation cohort ranged from −12 to 62, with a mean of 2.13 (standard deviation: 6.85). The distribution of the index values in the validation cohort is presented in Fig. 1. The values with small numbers of patients were grouped to include at least 1 % of the patients. The observed death rates for the values and their 95 % confidence intervals (CI) and the expected death rates are also presented.

**Fig. 2:**
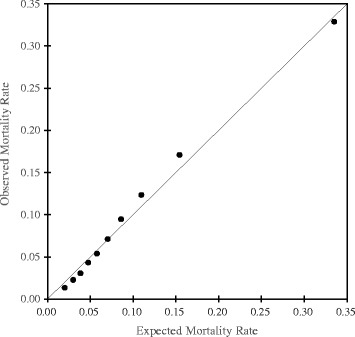
Calibration plot of the model predicting in-hospital death in the validation cohort

The *c*-statistics for models 1, 2, 3, and 4 were 0.701 (95 % CI: 0.698, 0.705), 0.758 (95 % CI: 0.755, 0.761), 0.675 (95 % CI: 0.672, 0.679), and 0.767 (95 % CI: 0.764, 0.770), respectively. The IDI for the comparison of model 4 with model 3 was 0.0700 (95 % CI: 0.0682, 0.0719), representing improved discrimination in model 4. The *ω*-statistic in model 4 was 1.09 (95 % CI: 1.03, 1.17), representing a slightly larger contribution of the index compared with the other four variables combined. Model 4 was well-calibrated, as shown by the Hosmer–Lemeshow calibration plot presented in Fig. 2.

**Fig. 3:**
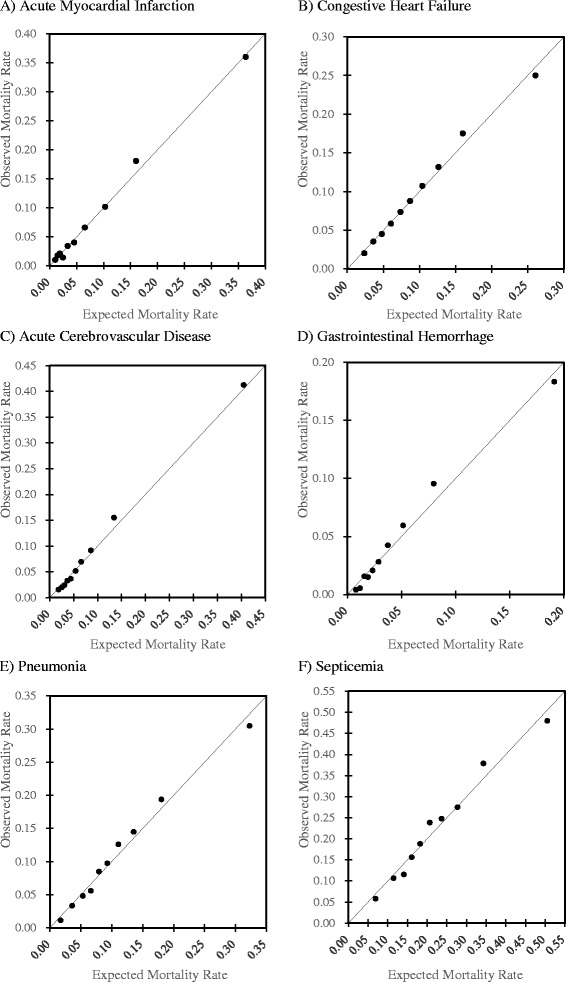
Calibration plots of models predicting in-hospital death in the validation cohort for six admissionprecipitating diagnoses (**a**, acute myocardial infarction; **b**, congestive heart failure; **c**, acute cerebrovascular disease; **d**, gastrointestinal hemorrhage; **e**, pneumonia; **f**, septicemia)

The mortality rates and *c*-statistics of the subgroups of patients are presented in Table 4. The model was well-calibrated for each diagnosis, as shown in Fig. 3. The model was also well-calibrated for other subgroups (data not shown).

**Table 4:** Results of subgroup analyses

	n	Mortality rate	*c*-statistic	95 % CI
Admission-precipitating diagnosis				
Acute myocardial infarction	10 176	8.6 %	0.821	0.806 – 0.836
Congestive heart failure	56 927	9.8 %	0.709	0.702 – 0.716
Acute cerebrovascular disease	90 071	8.6 %	0.801	0.796 – 0.807
Gastrointestinal hemorrhage	34 429	4.7 %	0.791	0.780 – 0.801
Pneumonia	68 386	11.0 %	0.733	0.727 – 0.739
Septicemia	9342	22.4 %	0.699	0.686 – 0.711
Admission by ambulance				
No	166 979	6.5 %	0.769	0.764 – 0.773
Yes	102 327	14.3 %	0.744	0.740 – 0.748
Referral from another institution				
No	161 494	9.9 %	0.769	0.766 – 0.773
Yes	107 808	8.8 %	0.762	0.757 – 0.767
Hospital type				
Non-academic	225 479	9.6 %	0.767	0.764 – 0.770
Academic	34 162	8.3 %	0.759	0.749 – 0.769

CI – confidence interval

## Discussion

Using the Diagnosis Procedure Combination nationwide administrative database of acute-care hospitals, we derived and internally validated a severity index for inpatients that utilizes procedure records to predict in-hospital death. In the patients with the six diseases examined, the index was widely distributed, and the model combining the severity index with age, sex, and CCI predicted in-hospital death well (*c*-statistic: 0.767).

We used procedures performed on the day of admission as indicators of severity on admission, and extracted 19 commonly performed procedures, diagnostic and therapeutic, that were significantly associated with in-hospital death or survival. The characteristics of the procedures differed widely, from routinely performed procedures (e.g. blood examinations) to those reflecting critically ill conditions (e.g. intratracheal intubation). This difference was represented in the weights given to each procedure, ranging from −3 to 23. The weights represented the strength of association between each procedure and death, relative to an increase in the CCI. The weighted numbers of the performed procedures were then summed into an index with a possible range of −13 to 69.

The mortality-predicting model with only diagnosis, age, sex, and CCI (model 3) had a fair discriminating ability (*c*-statistic: 0.675), and there was a significant improvement on the model performance when the severity index was added (IDI: 0.0700; *c*-statistic of model 4: 0.767). Furthermore, in model 4, the index contributed to the prediction of death more than all the other variables combined (*ω*-statistic: 1.09). These results represent the importance of the severity index for predicting mortality. To our knowledge, this is the first study to examine a mortality prediction model with commonly used procedures and medications, and the results suggest the usability of procedure records for risk adjustment.

Similar to other studies [12, 13], we chose six high-impact medical conditions (acute myocardial infarction, congestive heart failure, acute cerebrovascular disease, gastrointestinal hemorrhage, pneumonia, septicemia) as the target diseases. Although the single model had a good discriminating ability and was well-calibrated across various subgroups, the *c*-statistics ranged from 0.70 for septicemia to 0.82 for acute myocardial infarction. A previous study that used demographics, admission diagnosis, comorbidity-based score, and laboratory-based score as variables had similar results, in which the *c*-statistics were ≥0.80 for 29 admission diagnoses, 0.71–0.80 for 13 admission diagnoses, and <0.70 for two admission diagnoses [14]. Use of the same model for various primary illnesses may result in variable predictive ability across diagnoses because the effects of procedures on mortality may differ across diagnoses. In addition, the main diagnosis or main therapeutic procedure themselves are predictors of mortality [14, 15, 20]. Therefore, care should be taken when comparing these results with models derived separately for different diagnoses, which often yield higher *c*-statistics [12, 13, 16–19].

Although comorbidities recorded in administrative databases have provided fairly good predictions of mortality, there have been concerns that diagnoses may reflect complications instead of comorbidities [6–10]. The use of numerical laboratory data is one method suggested by researchers, and high model *c*-statistics of >0.8 were observed [12–16]. When available, laboratory data provide precise information about patient severity on admission and help to improve the model performance. However, implementation of an administrative database with laboratory data requires considerable cost and effort, and previous studies were thus confined to regional databases. In contrast, our study was conducted using a preexisting nationwide administrative database, and the procedures added considerable predictive ability to a model using demographics and comorbidities. The method presented here could be useful for similar databases with procedure data. For databases without procedure data, we recommend adding such data because it is relatively inexpensive and useful for mortality prediction.

Our study has several strengths. First, it was conducted using a nationwide database, and included patients of all ages treated in hospitals with different characteristics in all areas of Japan. Second, chronological information was considered in the diagnoses and procedures. We used the “admission-precipitating diagnosis” for case identification and the “comorbidities present on admission” for comorbidities. Similar to the use of “present on admission” codes in the previous US studies [12, 28] and “diagnosis-type indicator” codes in the previous Canadian studies [9, 29], our method prevents the misclassification of complications occurring during hospitalization as main diagnoses or comorbidities. Likewise, the information regarding dates of performance of procedures enabled the extraction of procedures performed on the day of admission. Third, we used the aspects of whether or not the procedures were performed as variables. Although procedure data are not as objective as automatically-recorded laboratory data, we believe that the validity of procedure data is higher than that of recorded diagnoses.

This study has several limitations. First, we only examined six medical conditions. It is unknown whether the severity index for inpatients developed in the present study is applicable to patients hospitalized for other conditions. Second, we excluded patients with critically ill conditions on the day of admission, because we expected that the associations of procedures with mortality would be different in these patients. Although some severe patients, such as intubated patients and those on catecholamines, were treated in general wards, as sometimes occurs in Japan [30, 31], and were thus included in the analysis, the issue of whether the index is valid for most critically ill patients, e.g. those admitted to the intensive care unit, requires further examination. Third, we limited the drugs to catecholamines and vasopressin, but other treatments such as intravenous fluids and antibiotics could also represent the severity on admission. Fourth, each admission was considered independent in the analyses. Better mortality prediction may be possible when clustering within patient and within site is taken into account. Also, the variance within each procedure, e.g., numbers or types of tested items within a blood test, was not accounted for. Last, the study was conducted in Japan using procedure codes in the Japanese fee reimbursement system, and the use of this index in other countries with different routine practices and coding systems will require appropriate conversions.

## Conclusion

The newly developed severity index for inpatients using procedure records predicted in-hospital death well. Further validating research should lead to its application to risk adjustment.

## Additional file

Additional file 1:
**Procedures examined in the study.**
