# Supplementary material for: Procedure-based severity index for inpatients: development and validation using administrative database
Source: BMC Health Serv Res. 2015 Jul 8;15:261. doi: 10.1186/s12913-015-0889-x (PMC4495704; doi:10.1186/s12913-015-0889-x)
Supplement: Additional file 1: — Procedures examined in the study. [file 12913_2015_889_MOESM1_ESM.doc]

| **Additional file. Procedures examined in the study** | | | | |
| --- | --- | --- | --- | --- |
| Procedures used as exclusion criteria | | | | |
| Code | Procedure | | Code | Procedure |
| A301, A301-2 | Intensive care unit admission | | K601 | Cardiopulmonary bypass |
| J046, K545 | Cardiopulmonary resuscitation | | K602 | Extracorporeal membrane oxygenation |
| J047 | Electrical cardioversion | | K603 | Ventricular assist device |
| Procedures examined for index development | | | | |
| Code | Procedure | Examples of items  Procedure | | |
| D000 | Urine tests (general) | pH, specific gravity, protein, glucose, occult blood, urobilinogen | | |
| D001 | Urine chemistry tests | Protein (quantitative), vanillylmandelic acid, Bence Jones protein | | |
| D002, D002-2 | Urine microscopy | Red blood cells, white blood cells, epithelial cells, casts | | |
| D003 | Stool tests | Ova and parasites, fat, hemoglobin, chymotrypsin | | |
| D004 | Examination of collected body fluids | Cerebrospinal fluid, gastric acid secretion, Huhner test | | |
| D005 | Hematology tests | Hemoglobin, white blood cell count, platelet count | | |
| D006 | Coagulation tests | Prothrombin time, fibrin degradation product, D-dimer | | |
| D007 | Blood chemistry tests | Sodium, potassium, chloride, total protein, total bilirubin | | |
| D008 | Endocrinology tests | Thyroid-stimulating hormone, follicle-stimulating hormone | | |
| D009 | Tumor markers | Carcinoembryonic antigen, alpha-fetoprotein, prostate-specific antigen | | |
| D011 | Immunohematology tests | Blood type, Coombs test, irregular antibodies, antiplatelet antibodies | | |
| D012 | Infectious disease immunology tests | Human immunodeficiency virus antibodies, anti-streptolysin O | | |
| D013 | Hepatitis virus tests | Hepatitis B surface antigen, hepatitis C antibodies | | |
| D014 | Autoantibody tests | Rheumatoid factor, antinuclear antibodies, cold agglutinin | | |
| D015 | Plasma protein immunology tests | C-reactive protein, complement components, glucose 6-phosphatase | | |
| D016 | Cellular function tests | B cell surface immunoglobulin, lymphocyte stimulation test | | |
| Procedures examined for index development | | | | |
| Code | Procedure | | Code | Procedure |
| D017 | Bacterial microscopy | | E200 | Computed tomography scan |
| D018 | Bacterial culture | | E202 | Magnetic resonance imaging |
| D019 | Bacterial drug susceptibility tests | | G002 | Arterial infusion |
| D019-2 | Fungal drug susceptibility tests | | G004 | Peripheral intravenous infusion |
| D020 | Acid-fast bacilli culture | | G005 | Central venous infusion |
| D021 | Acid-fast bacilli identification tests | | G005-2 | Central venous catheter insertion |
| D022 | Acid-fast bacilli drug susceptibility tests | | G005-3 | Peripherally inserted central catheter insertion |
| D023 | Nucleic acid amplification tests | | G005-4 | Cuffed central venous catheter insertion |
| D200 | Spirometry | | G006 | Infusion via central venous port |
| D203 | Alveolar function tests | | G008 | Intraosseous infusion |
| D206 | Cardiac catheterization | | G009 | Intrathecal injection |
| D208 | Electrocardiogram | | G011 | Endotracheal injection |
| D215 | Ultrasound imaging | | J000 | Wound treatment |
| D220 | Heart rate/respiration monitoring | | J001 | Burn treatment |
| D222 | Continuous blood gas monitoring | | J001-4 | Decubitus treatment |
| D223 | Pulse oximetry | | J002 | Drainage |
| D224 | Capnometry | | J008 | Thoracentesis |
| D225 | Invasive arterial pressure measurement | | J010 | Abdominal paracentesis |
| D225-2 | Continuous noninvasive arterial pressure measurement | | J018 | Sputum suction |
| D226 | Central venous pressure measurement | | J18-2 | Bronchial secretion suction under bronchoscope |
| D230 | Pulmonary artery pressure measurement | | J019 | Thoracic drainage tube insertion |
| D235 | Electroencephalography | | J020 | Gastric drainage tube insertion |
| D403, J007 | Cervical/thoracic/lumbar puncture | | J021 | Pleural drainage tube insertion |
| D404, J011 | Bone marrow aspiration | | J022 | Enema |
| E002 | Radiography | | J022-2 | Fecal disimpaction |
| E003 | Contrast agent infusion | | J023 | Injection via bronchial catheter |
| E100 | Scintigraphy | | J024 | Oxygen administration |
| E101 | Single photon emission computed tomography | | J026 | Intermittent positive pressure ventilation |
| E101-2 | Positron emission tomography | | J026-2 | Non-invasive positive pressure ventilation |
| E101-3 | Positron emission tomography/computed tomography | | J034 | Long tube insertion |
| **Additional file. Procedures examined in the study (continued)** | | | | |
| Procedures examined for index development | | | | |
| Code | Procedure | | Code | Procedure |
| J038 | Hemodialysis | | J048 | Pericardiocentesis |
| J038-2 | Continuous hemodiafiltration | | J049 | Esophageal balloon tamponade |
| J039 | Plasma exchange | | J050 | Bronchial washing |
| J041 | Hemoadsorption | | J051 | Gastric lavage |
| J042 | Peritoneal dialysis | | J058 | Bladder puncture |
| J043-3 | Stoma care | | J063 | Urinary catheter insertion |
| J043-4 | Gastrostomy tube exchange | | J064 | Temporary urinary catheterization |
| J043-5 | Urostomy catheter exchange | | J120 | Nasogastric tube insertion |
| J044 | Intratracheal intubation | | K386 | Tracheotomy |
| J044-2 | Transcutaneous/esophageal cardiac pacing | |  |  |
